# Utilization of carrageenan, citric acid and cinnamon oil as an edible coating of chicken fillets to prolong its shelf life under refrigeration conditions

**DOI:** 10.14202/vetworld.2016.166-175

**Published:** 2016-02-16

**Authors:** Anshul Kumar Khare, Robinson J. J. Abraham, V. Appa Rao, R. Narendra Babu

**Affiliations:** Department of Livestock Products Technology (Meat Science), Madras Veterinary College, Tamil Nadu Veterinary and Animal Sciences University, Chennai - 600 007, Tamil Nadu, India

**Keywords:** carrageenan, chicken breast/fillets, cinnamon oil, edible coating, spraying/brushing/dipping

## Abstract

**Aim:**

The present study was conducted to determine efficacy of edible coating of carrageenan and cinnamon oil to enhance the shelf life of chicken meat stored under refrigeration conditions.

**Materials and Methods:**

Chicken breast was coated with carrageenan and cinnamon oil by three methods of application *viz*., spraying brushing and dipping. The coated meat was evaluated for drip loss, pH, thiobarbituric acid number (TBA), tyrosine value (TV), extract release volume (ERV), Warner-Bratzler shear force value (WBSFV), instrumental color, microbiological, and sensory qualities as per standard procedures.

**Results:**

There was a significant difference observed for physicochemical parameters (pH, TBA, TV, ERV, drip loss and WBSFV) and microbiological analysis between storage periods in all the samples and between the control and treatments throughout the storage period but samples did not differed significantly for hunter color scores. However, there was no significant difference among three methods of application throughout the storage period though dipping had a lower rate of increase. A progressive decline in mean sensory scores was recorded along with the increase in storage time.

**Conclusion:**

The carrageenan and cinnamon edible coating was found to be a good alternative to enhance the shelf life of chicken meat under refrigeration conditions. It was also observed from study that dipping method of the application had comparatively higher shelf life than other methods of application.

## Introduction

Poultry breast (fillets) is a very popular food commodity and its consumption has increased over the last decades in Indian subcontinent. Broiler meat production in India is nearly 2.47 million tonnes [1] with growth rate of 6%. Exports of poultry products are currently 5,56,698 millon tonnes worth about Rs. 6512.1 millions with a growth rate of 7%. [2]. The processed meat industry is growing even much faster, at about 20%.

With the proliferation of different sources of media and use of these information media for education and awareness on transfer of technologies on good quality complete proteinaceous foods, consumption of animal origin foods are increasing. To have essential amino acids in the diet of human beings, supply of about 20-25% of total daily protein needs to be made through good quality proteinaceous foods of animal origin.

However, under Indian conditions meat and meat products are also prone to lipid oxidation because of high ambient temperature and lack of cold chain which eventually leads to spoilage of meat products [3]. Therefore, development of conditions such as edible coating could be a good option that can increase the shelf life of meat and its product. Edible coatings have been particularly considered in food preservation, because of their capability for improving global food quality by preventing quality loss such as shrinkage, oxidative off-flavors, microbial contamination, and discoloration in meat and meat products [4]. The edible coating is also defined as thin layers of edible materials, are usually applied as a liquid of varying viscosity to the surface of food product by spraying, dipping, brushing or other methods. Polysaccharides, proteins, and lipids are the main polymeric ingredients used to produce edible coating [5,6]. Polysaccharides are similar to hydrophilic materials; their polarity determines their poor barrier to water vapor as well as sensitive to moisture, which affects their functional properties [7]. Carrageenan, a naturally occurring anionic sulfated linear polysaccharides extracted from certain red seaweed [8] of the Rhodophyceae family. Carrageenan can function as a bulking agent, carrier, emulsifier, gelling agent, glazing agent, humectants, stabilizer, or thickener [9].

Cinnamon (*Cinnamomum zeylanicum* or *Cinnamomum verum*) belongs to the Lauraceae family and is an importanttraditional herbal medicine that is widely distributed in China,Vietnam, Sri Lanka, Madagascar, Seychelles and India [10]. It contains large quantities of terpenes andaromatic compounds specifically, cinnamaldehyde [11]. It is used worldwide as a food additive, flavoring agent and hasgood antioxidant and antimicrobial potential and it is considered “Generally Recognized as Safe (GRAS)” by US Food and Drug Administration [12-15]. The possibility of incorporating active compounds (antimicrobials, antioxidants, nutraceuticals, flavors, colorants) in polymeric matrices is one of the main advantages of coatings [16].

Citric acid is a hydroxy tricarboxylic acid produced naturally by various plants. It is water soluble, approved for direct addition to multiple foods, is affirmed as GRAS and is approved for use in the manufacture of fresh and processed meats and poultry at concentrations specific to its purpose. It has antimicrobial as well as tenderizing effect in meat and meat products [17]. The literature related to the application of hydrocolloids such as carrageenan and essential oil such as cinnamon oil as edible coating is very scanty and also no previous literature available regarding comparative study of three methods of application *viz*. spraying, brushing and dipping of coating chicken fillets. Carrageenan, citric acid, and cinnamon oil coating showed antimicrobial and antioxidant activity. Therefore, this study provides innovative and novel approach for extending shelf life of chicken meat under refrigeration conditions and this study also helpful to determine suitable method of application of coating among three methods *viz*. spraying, brushing and dipping.

The aim of this study was to evaluate the efficiency of edible coating of carrageenan incorporated with citric acid and cinnamon oil on the shelf life of chicken fillets stored under refrigerated conditions and also to select suitable method of application out of three methods *viz*. spraying, brushing and dipping.

## Materials and Methods

### Ethical approval

Permission of Animal Ethics Committee of Madras Veterinary College was taken for slaughter of experimental birds.

### Source of meat

Meat samples required for the experiments were obtained from broilers slaughtered as per standard procedure in the experimental slaughterhouse of Department of Livestock Products Technology (Meat Science) at Madras Veterinary College, Chennai-7, Tamil Nadu. The breast portion of the dressed carcasses (boneless skinless breast) after removal of all separable connective tissues, fat, skin, fascia, and blood vessels were used for edible coating. Analytical grade chemicals and media, required for analyzing the coated meat were procured from standard firms like SRL, Fisher Scientific, CDH, HiMedia, Sigma-Aldrich, etc. Cinnamon bark oil was procured from Plant Lipids Pvt. Cochin, Kerala.

### Preparation of coating solution

A coating solution was prepared by adding carrageenan and potassium chloride (1%) in ratio of 4:1 and citric acid was added at a level of 0.5% w/v and coating solution is heated at 60°C. This coating solution was followed with 0.05% cinnamon oil addition and proper mixing (carrageenan citric acid and cinnamon oil were selected on the basis of preliminary trials and previous literature available) and then divided into three parts 100 ml each for each for spraying and brushing and rest of 800 ml for dipping. pH of the coating solution were 7.56 (without citric acid) and citric acid incorporated coating solution had pH of 3.88-4.

### Methods of application

#### Spraying

Spraying was performed using hand sprayer, 50-100 ml coating solution was filled in sprayer then it was uniformly sprayed all over the breast (500-600 g). After deboning, spraying was also done on the back side which remained unsprayed.

#### Brushing

Boneless skinless breast (500-600 g) was brushed with coating solution (50 ml) using brush (4 cm×2 cm) uniformly and once after deboning on the remaining part.

#### Dipping

Dipping was done in a vessel containing 700-800 ml of coating solution. In this vessel, breast is dipped for 30 s after that draining of coating solution from breast was done for 30 s.

#### Packaging of coated meat

The meat was deboned and 60 g of meat packaged separately for control, spraying, brushing, and dipping stored under refrigeration temperature at 4±1°C (Samsung, India).Low-density polyethylene and polyester propylene laminated plastic bags of 200 Gauge in natural color were procured from reputed firms (Jeyam Plastics, Chennai) and used for aerobic packaging of coated chicken meat. 5 g (control and three treatments) of meat was packed separately in small lockable polythene bags (10 g size) for microbiological analysis. The coated meat samples were drawn at alternate days (1^st^, 3^rd^, 5^th^, and 7^th^) and analyzed for various physicochemical, microbiological and sensory attributes. Economics of coating solution was also estimated (Table-1).

**Table-1:** Economics of coating of chicken breast meat with carrageenan and cinnamon oil coating solution.

Characteristics	Carrageenan
Quantity/breast (500-600 g)	50-100 ml (S/B) 600 ml (D)
Name of company	HiMedia
Cost of pack	Rs. 690/100 g
Cost of coating solution	Rs. 7/100 ml (max)

S=Spraying, B=Brushing, D=Dipping - Methods of application of coating solution

### Analytical procedures

The pH of chicken meat was determined [18] with digital pH meter equipped with a combined glass electrode (Digisun Electronics System Model No. 2001). The estimation of water-holding capacity (WHC) of the coated chicken meat samples were carried out by adopting the filter paper press method recommended by Grau and Hamm [19,20] with slight modifications. A extract release volume (ERV) was determined by modified method of Pearson [21]. Drip loss was estimated as per the method outlined by Somers *et al.*, [22]. Tyrosine value (TV) and thiobarbituric acid (TBA) value were determined by the modified method by Strange *et al.*, 1977 [23]. The ability to scavenge 1, 1 diphenyl-2picrylhydrazyl radical by added antioxidants in coating solution (Table-2) was estimated following the method of Khare *et al.*, [24] with slight modifications. The polyphenol content (Table-2) was quantified by Folin–Ciocalteau's reagent and was expressed as gallic acid equivalents [24]. Warner-Bratzler shear force value (WBSFV) of frozen chicken breast meat was determined using Warner-Bratzler shear (G.R. Electric Manufacturing Co., Manhattan, USA). Color changes were measured using a MiniScan XE Spectrophotometer (Hunter Associates Laboratory, Reston, Virginia, USA), standard plate counts (SPC) in the samples were enumerated following the methods as described by American Public Health Association [25]. A six-member experienced panel of judges consisting of faculty and postgraduate students of Madras Veterinary College, Chennai-7 evaluated the samples for the attributes of color, odor and general appearance using 9 points descriptive scale [26] for color and general appearance while 10 point scale for odor.

**Table-2:** DPPH and total phenolic content of coating solution.

DPPH (% scavenging activity)	32.68
Total phenolics (gallic acid equivalent mg/g)	0.93

DPPH=1, 1 diphenyl-2picrylhydrazyl

### Statistical analysis

Data were analyzed statistically on “SPSS-16.0” software package as per standard methods [27]. Samples were drawn for each parameter, and the experiment was replicated six times (n=6). Sensory evaluation was performed by a panel of six trained panelist. Data were subjected to one-way analysis of variance, homogeneity test and Duncan's multiple range test for comparing the means to find the effects between treatment and between storage periods.

## Results and Discussion

### Physico-chemical parameters

#### pH

There was no significant difference (p>0.05) in pH values in between the treatments during 1^st^ and 3^rd^ day of storage, whereas a significant difference (p<0.05) was observed during 5^th^ and 7^th^ day of storage (Table-3). There was highly significant increase (p<0.01) in pH with increase in storage period in all the samples. Control samples had the highest values followed by spraying, brushing, and dipping throughout storage period. Coated meat samples had comparatively lower values than control. This might be attributed to the addition of citric acid in coating solution. Similar, increase in pH during storage period was reported by Sinhamahapatra *et al.*, [28] in broiler carcasses dipped and sprayed with decontaminants (lactic acid, acidified sodium chlorite [ASC] solution and chlorine solution). However, Petrou *et al.*, [29] observed no significant difference in pH of chicken fillets dipped in chitosan and oregano oil throughout storage period.

**Table-3:** Mean±SE values of physico-chemical properties (pH, ERV and water holding capacity, TBA, TV, drip loss and WBSFV) of carrageenan, potassium chloride, citric acid, and cinnamon oil coated chicken meat stored at 4±1°C.

Days	Methods of application				

	Control	Spraying	Brushing	Dipping	F value
pH					
1^st^	5.82±0.06^aA^	5.91±0.08^aA^	5.88±0.04^aA^	5.94±0.05^aA^	0.68^NS^
3^rd^	6.10±0.13^bA^	6.04±0.08^aA^	5.96±0.05^aA^	5.95±0.09^aA^	1.20^NS^
5^th^	6.15±0.09^bB^	6.28±0.07^bB^	6.16±0.04^bAB^	6.03±0.03^abA^	4.62*
7^th^	6.59±0.07^cC^	6.38±0.04^bBC^	6.29±0.07^bAB^	6.20±0.04^bA^	6.26**
F value	11.66**	9.54**	12.29**	4.26*	
ERV					
1^st^	18.25±0.48^cA^	20.33±0.82^cB^	19.25±0.17^cAB^	20.00±0.55^bB^	2.76^NS^
3^rd^	15.42±0.78^bA^	18.42±0.37^bB^	18.33±0.49^bB^	19.67±0.85^bB^	7.54**
5^th^	14.42±0.58^abA^	15.83±0.78^aAB^	16.92±0.54^aB^	17.25±0.91^aB^	3.15*
7^th^	12.75±0.48^aA^	14.00±0.46^aB^	14.00±0.46^aC^	16.33±0.17^aC^	21.92**
F value	15.14**	18.95**	26.96**	6.88**	
WHC					
1^st^	1.72±0.09^aA^	1.70±0.16^aA^	1.97±0.21^aA^	2.07±0.09^aA^	1.53^NS^
3^rd^	2.13±0.08^bA^	2.13±0.14^bB^	2.25±0.17^bB^	2.10±0.26^aB^	1.66**
5^th^	2.48±0.07^cA^	2.30±0.05^bcA^	2.50±0.17^bcA^	2.32±0.19^aA^	0.62^NS^
7^th^	2.70±0.14^cA^	2.60±0.08^cA^	2.80±0.25^cA^	3.03±0.13^bA^	1.29^NS^
F valve	18.34**	9.77**	3.03**	6.21**	
TBA number					
1^st^	0.05±0.009^aB^	0.02±0.003^aA^	0.03±0.006^aA^	0.02±0.005^aA^	4.15*
3^rd^	0.09±0.015^bB^	0.05±0.004^bA^	0.05±0.003^abA^	0.05±0.008^bA^	5.53**
5^th^	0.10±0.007^bcB^	0.06±0.007^cA^	0.07±0.010^bAB^	0.08±0.012^bcAB^	0.39^NS^
7^th^	0.13±0.005^cB^	0.09±0.006^dA^	0.11±0.020^aAB^	0.09±0.003^cA^	2.71^NS^
F value	0.264**	25.228**	8.539**	16.133**	
TV (mg/100 g)					
1^st^	2.84±0.30^aB^	2.50±0.16^aAB^	1.93±0.19^aA^	2.16±0.22^aAB^	3.140*
3^rd^	3.97±0.26^bB^	2.27±0.15^aA^	2.43±0.17^aA^	3.37±0.24^bB^	14.234**
5^th^	4.64±0.28^bB^	4.00±0.27^bcAB^	3.52±0.26^bA^	3.17±0.26^bA^	5.640**
7^th^	4.80±0.27^bC^	4.57±0.19^cBC^	4.07±0.20^bAB^	3.59±0.19^bA^	6.136**
F value	10.35**	15.812**	16.74**	14.66**	
Drip loss (%)					
1^st^	3.45±0.15^aB^	3.00±0.09^aAB^	3.22±0.12^aAB^	2.94±0.19^aA^	2.52^NS^
3^rd^	4.57±0.21^bAB^	5.38±0.41^bB^	4.49±0.29^bAB^	4.44±0.19^bA^	2.35^NS^
5^th^	5.61±0.25^cA^	6.05±0.19^cA^	6.29±0.22^cAB^	6.66±0.16^cB^	3.26*
7^th^	7.33±0.07^dB^	6.35±0.14^dA^	6.80±0.09^cAB^	7.34±0.58^cB^	4.33*
F value	78.313**	75.47**	69.85**	26.70**	
WBSF (kg/cm^2^)					
1^st^	0.87±0.06^aC^	0.56±0.02^aA^	0.88±0.02^aB^	0.71±0.06^aC^	10.99**
3^rd^	1.02±0.05^abB^	0.69±0.03^abA^	1.05±0.02^bB^	0.73±0.06^aA^	16.78**
5^th^	1.08±0.07^bB^	0.74±0.04^bA^	1.06±0.05^bA^	0.82±0.06^aB^	9.07**
7^th^	1.18±0.05^bC^	0.83±0.05^bA^	1.18±0.02^cC^	1.01±0.05^bB^	12.39**
F value	4.83*	14.73**	9.32**	5.27**	

Means bearing different superscript between rows a, b, c and between columns A, B, C differs significantly (p<0.05)

* Indicates significant value (p<0.05)

** Highly significant value (p<0.01). NS=Non significant, ERV=Extract release volume, WHC=Water holding capacity, TBA=Thiobarbituric acid, TV=Tyrosine value, WBSFV=Warner-Bratzler shear force value, SE=Standard error

#### ERV

ERV is an important indicator of spoilage in meat and its value decreases with storage period. During the initial days of storage, there was no significant difference (p>0.05) in between the treatments. The ERV values decreased significantly (p<0.01) with increase in storage period irrespective of different methods of application. Pearson [21] revealed that meat could be considered acceptable provided that the ERV is at least 17 ml. The ERV value of control was well below the acceptable limit during 3^rd^ day of storage. However, spraying, brushing, and dipping samples had ≤ 17 ml value during 5^th^ day of storage (Table-3). Dipping could be better method of application due to more viscous nature of carrageenan which coats the chicken breast effectively. These results were in agreement with Kandeepan and Biswas [30] who indicated that ERV values continuously decreased during refrigerated storage (23.5 ml on 0^th^ day to 14.3 ml on 7^th^ day) in buffalo meat. The decrease in ERV values could be attributed to increase in microbial count [31]. However, Sinhamahapatra *et al.*, [28] observed that spraying and dipping of chicken meat with various decontaminants (lactic acid, ASC solution and chlorine solution) did not cause any significant change in ERV values during storage.

#### WHC

There was a highly significant (p<0.01) difference in WHC between storage period in all the samples and WHC decreased significantly with storage period. However, no significant difference was observed in between the treatments in all the storage days except on the 3^rd^ day (Table-3). Coated meat sample had comparatively higher WHC compared to uncoated meat which could be attributed to lower moisture loss by application of carrageenan and addition of citric acid in coating solution which leads to decrease in pH. The results in the present study were in agreement with Ayadi *et al.*, [32] who revealed higher WHC in carrageenan added turkey meat sausages. Gault [33] proposed that the increased WHC of beef muscle at lower pH values was due to the increase in the net positive charges on the protein molecules and the osmotic pressure exerted by the presence of large amounts of organic acids to decrease pH. WHC of muscle foods increases when the pH is below the isoelectric point of the major myofibrillar proteins [33,34].

#### TBA number

**Figure-1:**
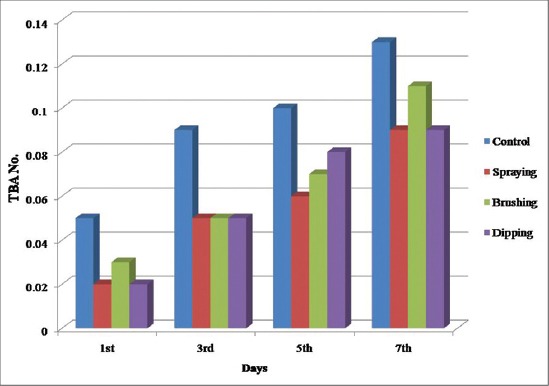
Thiobarbituric acid values of carrageenan, potassium chloride, citric acid, and cinnamon oil coated chicken meat stored at 4±1°C.

The TBA test has been widely used to estimate the extent of lipid oxidation in meat and meat products [35]. TBA value increased significantly with storage period in all the samples and during initial days of storage (Figure-1) there was highly significant difference in between control and treatments. However, no significant difference was observed during the 5^th^ and 7^th^ day of storage (Table-3). Coated meat irrespective of the methods of the application had slightly lower values than control samples. These results were in agreement with Wu *et al.*, [36] who opined that coating of precooked beef patties with carrageenan lowered the TBA values compared to control suggesting that the oxidation of precooked beef patties may be controlled to some extent by hydrocolloids like carrageenan.

Lower TBA values of coated meat might be attributed to the synergistic effect of carrageenan, cinnamon and citric acid on lipid peroxidation. These findings were in agreement with Pikul *et al.*, [37] who demonstrated a significant decrease in TBA values of various meats incorporated with butylated hydroxyanisole. Sheikh Dalia [38] also revealed lower TBA values in chicken breast meat coated with gum Arabic and plantago throughout the entire storage period of 21-day under refrigeration temperature. The result in the present study was in agreement with Qiu *et al.*, [39] who found lower TBA values in samples incorporated with chitosan, citric acid and licorice extract in Japanese fish fillets. Kamel [40] studied effect of mango kernel and cactus peel as edible coating on chicken breast meat and concluded that 25% cactus peels and 0.8% mango kernel showed the lowest TBA value of 0.421 mg malondialdehyde/kg compared to 1.30 mg malondialdehyde/kg in control after 2 weeks of storage

#### TV

The degree of autolysis and bacterial proteolysis in meat can be measured as TV which actually determines the quantity of amino acids, i.e., tyrosine and tryptophan present in an extract of meat. In the present study, TV increased significantly (p<0.05) with storage period and dipping samples showed the lowest TV followed by spraying and brushing while control had highest values (Table-3). Increase in TV of the control and treatment sample during storage period might be due to the increased microbial load and enhanced production of proteolytic enzymes in the late logarithmic phase of microbial growth; causing autolysis and bacterial proteolysis [41]. The results of the present study could be collated with the observation of Pearson [21], Strange *et al.*, [20]. The lower values in treatments could be attributed to antimicrobial activity of cinnamon oil and citric acid. Similar results were observed in duck patties stored at ambient and refrigeration temperature where TV increased significantly with storage period [42].

#### Drip loss

There was no significant difference (p>0.05) in drip loss in between the treatments during 1^st^ and 3^rd^ day of storage. However, control had higher values than treatments throughout the storage period. During 5^th^ and 7^th^ day of storage, there was significant (p<0.05) difference was observed in between the treatments (Table-3). Kester and Fennema [43] reported similar results with polysaccharide coating such as carrageenan which act as a moisture barrier when applied in food products such as meat. The results were also in agreement with Pearce and Lavers [44] who observed lower drip loss and higher shelf life in carrageenan dipped meat compared to uncoated meat. Drip loss increased significantly (p<0.01) throughout the storage period. This could be due to degradation of the protein resulting in expulsion of water that is expelled from intermyofibrillar spaces leading to drip. This result was in agreement with Lesiak *et al.*, [45] who found that longer the storage period greater the drip loss. Lee *et al.*, [46] also reported that broiler meat aged for 6 day had higher drip loss than that aged for 1 day. In the present study, at 5^th^ day of storage control samples had slightly lower value than treatments which could be due to high water loss during earlier storage period. However, at the end of the storage period (7^th^ day) control and dipping had the highest values followed by spraying and brushing. Lower drip losses in treatment could be attributed to antimicrobial activity of citric acid and cinnamon oil and its synergistic effect with carrageenan which had moisture barrier property/or reducing moisture loss.

#### WBSFV

There was a significant difference (p<0.05) in WBSF between the storage period and also in between the treatments. WBSFV increased significantly (p<0.01) with storage period, control had higher values followed by brushing, dipping and spraying (Table-3). WBSFV is inversely related to tenderness of meat. Lower WBSFV in treatment could be due to citric acid incorporation in coating solution. Komoltri and Pakdeechanuan [47] also observed lower shear force value in Golek chicken marinated with citric acid. Ke *et al.*, [48] suggested that tenderness is related to the pH of the muscle. They reported that Warner-Bratzler shear force decreased as muscle pH lowered to 3.52, and then shear force significantly increased as the pH was buffered back to pH 5.26. Many researchers have observed that the tenderness of muscle increased when the pH is below the isoelectric point of the major myofibrillar proteins [33,34].

#### Instrumental/hunter color

There was no significant difference in color between the storage periods, whereas a significant difference was observed in between the treatment during 1^st^ and 3^rd^ day of storage. Similar results were revealed by Machado de Melo *et al.*, [49] in refrigerated chicken meat in contact with cellulose acetate-based film incorporated with rosemary essential oil (20% and 50%, v/w) they found control samples and film incorporated with 50% rosemary essential oil had no significant variation with respect to the L*, a* and b* values between storage days and treatments. Coated meat sample had higher L* value than uncoated/control sample. The results were in contradiction to those reported by Tyburcy and Kozyra [50] who found lower L* value in carrageenan coated sausages. Chouliara *et al.*, [51] reported a decrease in L* parameter values in chicken breast meat with storage time in samples containing 0.1 ml/100 g oregano oil. There was no significant difference (p>0.05) in redness a* value observed between the treatments and between the storage period except on 3^rd^ day (Table-4). This might be due to antioxidant activity of cinnamon oil which prevent lipid oxidation and change in pigment color. Keokamnerd *et al.*, [52] reported a decrease in a* value in ground chicken meat during 12 days of storage. These results are in agreement with those of Rodríguez-Calleja *et al.*, [53] who found that a combination of high hydrostatic pressure, a commercial liquid antimicrobial edible coating and MAP did not affect color acceptability of chicken breast fillets. There was no significant difference between storage period and between treatments in yellowness value. There was a significant difference (p<0.05) in yellowness value between the treatments during the 3^rd^ day of storage. Coated meat samples revealed higher values than control which could be due to addition of cinnamon oil. This was in agreement with Lu *et al.*, [54] who also found higher yellowness value in fish fillets treated with cinnamon oil. Giatrakou *et al.*, [55] reported that b* (yellowness) values were varied with no specific pattern produced by any of the treatments (combination of thyme oil and chitosan) in a poultry product.

**Table-4:** Mean±SE values of instrumental/hunter color of carrageenan, potassium chloride, citric acid, and cinnamon oil coated chicken meat stored at 4±1°C.

Days	Methods of application				

	Control	Spraying	Brushing	Dipping	F value
L*value					
1^st^	56.93±1.15^aA^	61.49±1.34^aB^	62.30±1.05^aB^	62.94±1.24^aB^	5.14**
3^rd^	59.38±1.35^aA^	60.53±1.61^aB^	62.25±1.06^aB^	61.62±1.54^aB^	0.81**
5^th^	59.79±1.32^aA^	62.54±0.85^aA^	61.57±1.10^aA^	61.58±1.22^aA^	1.01^NS^
7^th^	60.02±1.63^aA^	61.48±1.31^aA^	63.27±0.75^aA^	62.89±1.46^aA^	1.23^NS^
F value	11.66**	9.54**	12.29**	4.26*	
a* value					
1^st^	18.25±0.48^cA^	20.33±0.82^cB^	19.25±0.17^cAB^	20.00±0.55^bB^	2.76^NS^
3^rd^	15.42±0.78^bA^	18.42±0.37^bB^	18.33±0.49^bB^	19.67±0.85^bB^	7.54**
5^th^	14.42±0.58^abA^	15.83±0.78^aAB^	16.92±0.54^aB^	17.25±0.91^aB^	3.15*
7^th^	12.75±0.48^aA^	14.00±0.46^aB^	14.00±0.46^aC^	16.33±0.17^aC^	21.92**
F value	15.14**	18.95**	26.96**	6.88**	
b* value					
1^st^	1.72±0.09^aA^	1.70±0.16^aA^	1.97±0.21^aA^	2.07±0.09^aA^	1.53^NS^
3^rd^	2.13±0.08^bA^	2.13±0.14^bB^	2.25±0.17^bB^	2.10±0.26^aB^	1.66**
5^th^	2.48±0.07^cA^	2.30±0.05^bcA^	2.50±0.17^bcA^	2.32±0.19^aA^	0.62^NS^
7^th^	2.70±0.14^cA^	2.60±0.08^cA^	2.80±0.25^cA^	3.03±0.13^bA^	1.29^NS^
F value	18.34**	9.77**	3.03**	6.21**	
ΔE value					
1^st^	58.54±1.90^aA^	64.49±1.12^aB^	65.06±1.09^aB^	65.48±1.24^aB^	5.60**
3^rd^	62.74±1.09^bA^	63.51±1.42^aB^	65.46±0.91^aB^	64.64±1.64^aB^	4.21**
5^th^	63.56±1.34^bA^	64.95±0.93^aA^	64.79±0.85^aA^	65.08±1.29^aA^	0.39^NS^
7^th^	62.96±1.09^bA^	64.62±1.59^aAB^	66.24±0.72^aAB^	66.88±1.08^aB^	2.27^NS^
F value	2.71^NS^	0.23^NS^	0.486^NS^	0.530^NS^	

Means bearing different superscript between rows a, b, c and between columns A, B, C differs significantly (p<0.05)

* Indicates significant value (p<0.05)

** Highly significant value (p<0.01). NS=Non significant, SE=Standard error

L*-Lightness, a*-redness, b*-yellowness and ΔE value-Total color change

Total color change (delta-E) value had significant (p<0.05) difference during 1^st^ and 3^rd^ day of storage. Control had lower value than treatments due to higher L* and b* value in treated samples. However, no significant difference (p>0.05) was observed during 5^th^ and 7^th^ day of storage (Table-4).

### Microbiological quality

#### SPC (log_10_cfu/gm)

**Figure-2:**
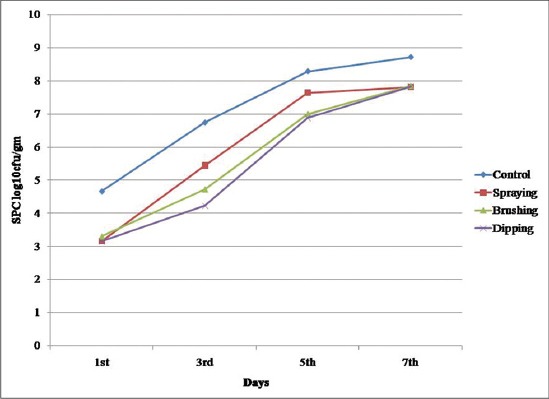
Standard plate count (log_10_cfu/g) of carrageenan, potassium chloride, citric acid, and cinnamon oil coated chicken meat stored at 4±1°C.

There was highly significant difference (p<0.01) in SPC between the treatments and in between storage period (Figure-2). During initial day of storage control had significantly (p<0.01) higher values compared to treatment. However, no significant difference was observed in between treatments during the 1^st^ day and SPC increased significantly (p<0.01) with storage period in all the samples (Table-5).

**Table-5:** Mean±SE values of SPC (log_10_ cfu/g) of carrageenan, potassium chloride, citric acid, and cinnamon oil coated chicken meat stored at 4±1°C.

Days	Methods of application				

	Control	Spraying	Brushing	Dipping	F value
SPC (log_10_ cfu/g)					
1^st^	4.66±0.30^aB^	3.16±0.10^aA^	3.31±0.14^aA^	3.15±0.09^aA^	16.84**
3^rd^	6.74±0.21^bC^	5.44±0.26^bB^	4.73±0.20^bAB^	4.23±0.10^bA^	19.44**
5^th^	8.29±0.10^cB^	7.64±0.24^cA^	7.00±0.23^cA^	6.88±0.31^cA^	6.52**
7^th^	8.72±0.12^cB^	7.81±0.27^cA^	7.85±0.28^dAB^	7.83±0.23^dA^	3.76*
F value	86.21**	91.83**	93.09**	113.76**	

Means bearing different superscript between rows a, b, c and between columns A, B, C differs significantly (p<0.05)

* Indicates significant value (p<0.05)

** Highly significant value (p<0.01). NS=Non significant, SPC=Standard plate count

SPC value on the 3^rd^ day in control sample was 6.74 log_10_cfu/g and it was very close to the maximum permissible limit of 7 log_10_cfu/g total viable count (TVC) for good quality fresh poultry meat as prescribed by ICMSF [56]. However, all other samples reached approxiamtely 7 log_10_cfu/g on 5^th^ day of storage and all the samples exceeded the limit on 7^th^ day of storage. Dipping sample had lower value throughout storage period than brushing and spraying. This might be attributed to antimicrobial activity of cinnamon and more viscous nature of carrageenan. Cinnamon oils contain high concentrations of trans-cinnamaldehyde, a well-known antimicrobial compound [57], and also contain linalool, eugenol and other phenolic compounds. Previous studies have also identified trans-cinnamaldehyde as the major antibacterial constituent of cinnamon oil [14].Similar results were revealed by Ojagh *et al.*, [58] in rainbow trout (*Oncorhynchus mykiss*) coated with chitosan (Ch) and cinnamon oil (Ch + C) under refrigerated storage (4±1°C) for a period of 16-day and found that coated sample exhibited good quality characteristics (lower microbial load) and higher shelf life.

Seol *et al.*, [59] also revealed the carrageenan film incorporated with ovotransferrin and ethylenediaminetetraacetic acid had antimicrobial activity than carrageenan alone and they found that chicken breast meat wrapped with carrageenan reached 7 log_10_cfu/g on 7^th^ day while treatments had nearly 5.23-6.91 log_10_cfu/g. Similarly, Shojaee-Aliabadi *et al.*, [60] observed that k-carrageenan film incorporated with plant essential oil had antimicrobial activity against most of the pathogenic microorganism. Olaimat *et al.*, [61] concluded that κ-Carrageenan/chitosan-based coatings containing 50 or 100 μl/g containing allyl isothiocyanate (AITC) reduced viable *Campylobacter jejuni* to undetectable levels on chicken breast after 5 day at 4°C, while 25 μl/g AITC or 200-300 mg/g mustard extract in coatings reduced *C. jejuni* numbers by 1.75-2.78 log_10_cfu/g.

#### Sensory attributes

Color scores decreased significantly (p<0.01) with storage period in all the samples and there was highly significant difference was observed between the storage period in all the samples and between samples throughout storage period (Figure-3a). Coated meat samples had higher color score than control, and this was in accordance with Cierach *et al.*, [62] who found higher color scores in carrageenan added sausages than control samples. On the last day of storage, all the samples had lowest values which might be attributed to higher microbial load which lead to change in color from pink to pale pink.

**Figure-3a:**
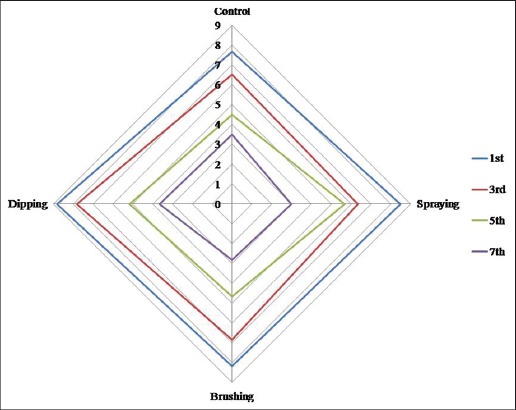
Sensory attributes (color) of carrageenan, potassium chloride, citric acid, and cinnamon oil coated chicken meat stored at 4±1°C.

Odor scores were not significant (p>0.05) during initial and final day of storage (Figure-3b). However, highly significant difference was observed during 3^rd^ and 5^th^ day of storage. Odor score reached unacceptable score on 3^rd^ day in control sample while on 5^th^ day in other treatments. At the end of storage (7^th^ day) no significant difference (p>0.05) was observed in odor score which could be correlated to higher bacterial load which leads to the production of sulfurous compounds/off odor. Similar results were obtained by Baston and Barna [63] who compared sensory scores (three point scale) of raw chicken leg and breast at refrigerated storage and revealed that after 1 week of storage breast meat had odor, skin color and slime formation score of 1.3, 2 and 2.3 while leg meat had 1.9, 2.3 and 2.2, respectively.

**Figure-3b:**
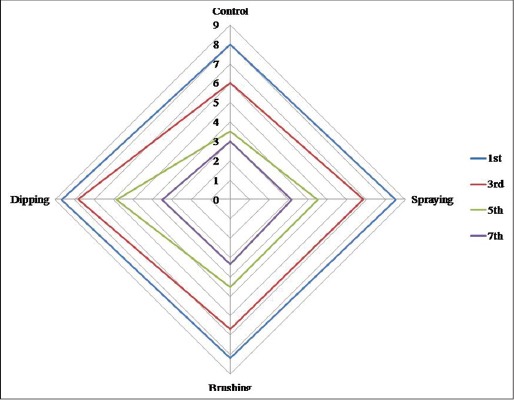
Sensory attributes (odor) of carrageenan, potassium chloride, citric acid, and cinnamon oil coated chicken meat stored at 4±1°C.

The antioxidant, antimicrobial and gas barrier effects by coating have been shown to minimize the oxidative effects, prolonging the product shelf life while maintaining quality. Mexis *et al.*, [64] reported that chicken meat treated with citrus extract and control had shelf life of 6 and 4 days, respectively, based on sensory scores and microbiological analysis. Del Rio *et al.*, [65] reported an increase in shelf life by 2 days for chicken legs after treatment with a solution of 2 ml citric acid/100 ml.

There was highly significant difference (p<0.01) was observed in general appearance scores (Figure-3c) in between the treatments throughout the storage period and between storage period in all the samples. It decreased significantly with storage period. During 7^th^ day of storage, there was no significant difference in between the treatments. Control had the lowest score followed by spraying, brushing and dipping sample throughout storage period. Data obtained from sensory panelist were in agreement with microbiological (TVC) quality results.

**Figure-3c:**
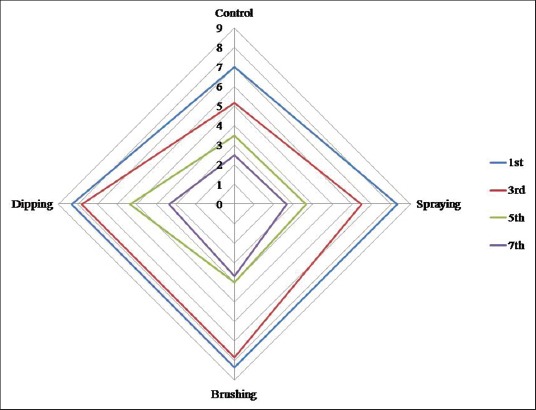
Sensory attributes (general appearance) of carrageenan, potassium chloride, citric acid, and cinnamon oil coated chicken meat stored at 4±1°C.

## Conclusion

The edible coating of carrageenan, cinnamon oil and citric acid can be used to enhance shelf life of chicken meat under chilled condition and dipping method is comparatively better than other methods of application.

## Authors’ Contributions

The study is the major component of the special problem of first author AKK. RJJA provided the guidelines during the work and corrected manuscript. VAR and RNB assisted in solving technical issues during program of work preparation and also give valuable suggestions throughout study. All the authors have read and approved the final manuscript.
